# Extracellular Vesicles as Surrogates for Drug Metabolism and Clearance: Promise vs. Reality

**DOI:** 10.3390/life13081745

**Published:** 2023-08-14

**Authors:** Anna Gagliardi, Gzona Bajraktari-Sylejmani, Elisabetta Barocelli, Johanna Weiss, Juan Pablo Rigalli

**Affiliations:** 1 Department of Clinical Pharmacology and Pharmacoepidemiology, Heidelberg University Hospital, Im Neuenheimer Feld 410, 69120 Heidelberg, Germany; 2 Department of Food and Drug, University of Parma, Parco Area delle Scienze 27/A, 43124 Parma, Italy

**Keywords:** ABC transporters, drug clearance, drug-metabolizing enzymes, drug transporters, exosomes, extracellular vesicles, liquid biopsy

## Abstract

Drug-metabolizing enzymes (DMEs) and transporters play a major role in drug efficacy and safety. They are regulated at multiple levels and by multiple factors. Estimating their expression and activity could contribute to predicting drug pharmacokinetics and their regulation by drugs or pathophysiological situations. Determining the expression of these proteins in the liver, intestine, and kidney requires the collection of biopsy specimens. Instead, the isolation of extracellular vesicles (EVs), which are nanovesicles released by most cells and present in biological fluids, could deliver this information in a less invasive way. In this article, we review the use of EVs as surrogates for the expression and activity of DMEs, uptake, and efflux transporters. Preliminary evidence has been provided for a correlation between the expression of some enzymes and transporters in EVs and the tissue of origin. In some cases, data obtained in EVs reflect the induction of phase I-DMEs in the tissues. Further studies are required to elucidate to what extent the regulation of other DMEs and transporters in the tissues reflects in the EV cargo. If an association between tissues and their EVs is firmly established, EVs may represent a significant advancement toward precision therapy based on the biotransformation and excretion capacity of each individual.

## 1. Introduction

Drug absorption, distribution, metabolism, and excretion (ADME) are important determinants of therapeutic efficacy and safety of pharmacological treatments. This is the result of a complex interplay of cellular and molecular factors, where drug-metabolizing enzymes and transporters play a major role. The expression and activity of these proteins are far from being static or easily predictable. The same applies to the pharmacokinetics of their substrates. A paradigm case is the treatment with digoxin, a drug with a particularly narrow therapeutic range (i.e., serum concentrations within 0.5–0.8 ng/mL) [1]. Instead, 20-fold differences among serum concentrations of different individuals have been achieved after administration of the same dose of the drug [2]. Therefore, without further monitoring, life-threatening situations of under- and overdosing can unavoidably take place. Advances in the field of pharmacogenetics provide useful information to estimate the potential drug metabolizing and excretion capacity of an individual. However, so far, they do not provide all the tools and the answers required to increase drug efficacy and safety [3]. Moreover, genotype analysis cannot provide tissue- and cell-specific information on drug-metabolizing enzymes (DMEs) and transporters. In this regard, new analytical and predictive tools are required. 

### 1.1. Drug Metabolism and Transport

#### 1.1.1. Drug Metabolism

Drug metabolism is conventionally distinguished into phase I and phase II reactions. Phase I metabolism, also known as phase I biotransformation, comprises hydrolysis, reduction, dealkylation, deamination, and oxidation reactions to facilitate drug conjugation and elimination. Oxidation reactions represent the most common form of phase I transformation, with most of the xenobiotics being metabolized by enzymes belonging to the cytochrome P450 superfamily [4,5].

Cytochrome P450 (CYP) enzymes are heme-containing proteins that mediate a variety of microsomal oxidoreduction reactions [4]. They are mainly expressed in the endoplasmic reticulum of hepatocytes, the primary site of drug metabolism. They are also highly expressed in epithelial cells of the small intestine, in tubular cells of the kidney, in the lungs, brain, and skin, along with endothelial cells, which contribute to the extrahepatic drug metabolism [6,7]. CYP1, CYP2, and CYP3 families and related isoenzymes are responsible for the biotransformation of most administered drugs and endogenous compounds [8]. From a pharmacological point of view, the bioavailability, efficacy, and toxicity of approximately 80% of the drugs depend on CYP-mediated metabolism. Particularly, CYP2C (CYP2C9, CYP2C19) and CYP3A (CYP3A4, CYP3A5) families are responsible for 25% and 50–60% of all clinically relevant drugs’ metabolism, respectively. Other involved isoenzymes are CYP1A2, CYP2E1, and CYP2D6 [9]. Importantly, more than one drug can be metabolized by the same isoform, increasing the probability of drug–drug interactions (DDI). In addition, situations of induction (mostly due to the increase in enzyme expression) and inhibition can occur [10,11]. Most inducers lead to an increase in enzyme expression and subsequent catalytic activity through the activation of different nuclear receptors (e.g., constitutive androstane receptor—CAR and pregnane X receptor—PXR) at the transcriptional level [12]. Regulation at the post-transcriptional and post-translational levels can also take place. In general, this enzymatic induction is a dose-dependent event that can persist even after a reduction in drug concentration and can negatively influence the pharmacokinetics of co-administered drugs. Due to CYP induction, either toxic, more active, or inactive compounds can be formed from the progenitor molecule. An example of a clinically significant interaction is given by the loss of activity of oral contraceptives when combined with rifampicin [13]. Moreover, the procarcinogen activity of some xenobiotics (e.g., polycyclic aromatic hydrocarbons—PAH) may be enhanced by the same mechanism [14,15]. In general, heterogeneous responses to medications can be highly attributed to interindividual variability in terms of the expression and regulation of phase I enzymes. Both factors need to be taken into consideration when deciding therapeutic strategies. These effects are even more important in poly-medicated patients.

Phase II reactions consist of the conjugation of endo- and xenobiotics as well as products of phase I reactions with endogenous compounds possessing different hydrophilic groups. In general, this promotes the secretion out of the cell and later excretion out of the organism. Also, conjugation results, in most cases, in the inactivation of the drug. Most phase II reactions involve the conjugation with glucuronic acid (catalyzed by UDP glucuronosyltransferases, UGTs) [16], glutathione (catalyzed by glutathione S-transferases, GSTs) [17], or sulfonate (catalyzed by sulfotransferases, SULTs) [18].

UGTs are divided into the families UGT1 and UGT2 and, although the liver is their main site of expression, they have also been described in other organs of highly pharmacological relevance, such as the gastrointestinal tract and the kidney. Reaction types and substrate specificity of UGTs have been reviewed elsewhere [16]. Although, in most cases, glucuronidation results in drug inactivation, drug activation has also been described. For instance, morphine-6-glucuronide exhibits a stronger analgesic effect than the parent drug [19]. UGTs can be modulated by endo- and xenobiotics; for example, via activation of PXR [20]. In this regard, alterations in the area under the plasmatic curve (AUC) of their substrates can be expected. This was clearly exemplified in the case of irinotecan and its active metabolite SN-38 in an experiment in rats exposed to the inducer phenobarbital. Here, a decrease in the AUC of SN-38 and a concomitant increase in the AUC of its glucuronide conjugate was observed with the risk of therapeutic failure [21].

GSTs catalyze the nucleophilic attack of glutathione to molecular regions with electronic gaps in endogenous and exogenous compounds, which results in their conjugation. A complete review of the classification, structure, localization, and substrates of GSTs was provided by Hayes and coworkers [17]. Changes in the exposure to toxic xenobiotics depending on the presence of specific polymorphisms in different GST genes have been described [22]. GSTs are also subject to regulation by xenobiotics [23].

Finally, SULTs catalyze the reactions of endo- and xenobiotics with 3′-phosphoadenosine 5′ phosphosulfate (PAPS), which result in the transfer of a sulfonate group to the accepting molecule. Pharmacologically relevant SULTs are expressed in the cytosol. Sulfonation usually leads to the inactivation of the parent compound [18]. An exception constitutes minoxidil, used in the treatment of alopecia, which is, instead, activated by SULT action [24]. Similar to other biotransformation enzymes, SULT expression can be positively and negatively regulated [18,25,26,27].

Altogether, biotransformation enzymes constitute important players in drug metabolism and, therefore, drug inactivation or activation. Regulation of phase I and phase II enzymes by a wide range of endogenous and exogenous compounds usually takes place in a coordinate manner (e.g., by the activation of nuclear receptors) and affects the pharmacokinetics of their substrates [20]. Thus, detecting and monitoring changes in the enzyme expression would be an important asset toward avoiding situations of drug toxicity or reduced therapeutic efficacy.

#### 1.1.2. Drug Transport

Multiple and different proteins are expressed at the plasma membrane level and throughout the intracellular organelles as well, with the role to transport substrates from extracellular compartments (e.g., blood) into the cells and remove substrates out of the cell, thus contributing to the control of cell homeostasis. Sugars, amino acids, nucleotides, metals, inorganic and organic ions, oligopeptides, and drugs are substrates of such transporters. Most transporter proteins belong to four main superfamilies: the ATP-binding cassette (ABC) transporters, the ATPases, the ion channels, and the solute carrier proteins (SLCs). Among the transporters belonging to the superfamily of the SLCs, the families SLC22 and SLCO are the most relevant [28]. The SLC22 family is subdivided into two main subfamilies: the organic anion transporters (OATs) and the organic cation transporters (OCTs). Within the OATs, for example, SLC22A6 (OAT1) mediates the basolateral uptake of anionic drugs in the renal proximal tubule, thus contributing to the excretion from the blood into the pro-urine [28]. Furthermore, SLC22A1 (OCT1), which is highly expressed in the basolateral membrane of the hepatocytes, plays a major role in the hepatic uptake of cationic drugs. OCT1-mediated drug uptake from the intestinal lumen has also been described [29]. Furthermore, the SLCO family comprises the organic anion transporter polypeptides (OATPs), which are, in turn, divided into subfamilies, OATP1 and OATP2 being the most relevant. In general, they mediate the basolateral uptake of anionic drugs into the hepatocytes and the apical uptake in renal proximal tubular cells and the enterocytes from the pro-urine and the intestinal lumen, respectively. In this regard, OATPs contribute to the hepatic excretion of a wide range of compounds. OATP1B1 and OATP1B3 play a major role in the liver uptake of statins and their hepatic clearance [30]. Also, loss-of-function of OATP1A/1B has been associated with increased bioavailability of methotrexate and paclitaxel in mice [31]. On the contrary, OATP-mediated reabsorption of drugs from the pro-urine decreases the renal clearance of substrates. This was observed, for example, for rosuvastatin in a model of OATP1A/1B null mice [32]. In addition, a decrease in the function of intestinal OATPs was related to decreased absorption of drugs, such as fexofenadine [33]. Notably, the above-mentioned opposite roles of OATP transporters, mediating intestinal drug absorption and hepatic clearance and counteracting renal clearance, constitute a particular challenge while investigating this subfamily of transporters. A similar case occurs for OCT1, which mediates intestinal absorption and hepatic clearance of many drugs. In this context, cell-specific markers aimed at distinguishing the renal, hepatic, and intestinal uptake activity would be highly beneficial in terms of deconstructing the tissue-specific contribution of these transporters to the global pharmacokinetic profile of drugs. 

Drug bioavailability, distribution, and elimination are also influenced by interaction with unidirectional efflux protein transporters highly expressed in the human body (e.g., intestine, liver, kidney, placenta, and blood–brain barrier). ATP-binding cassette-transporters (ABC transporters) constitute the most important protein superfamily responsible for transmembrane efflux transport. ABC transporters use energy from ATP hydrolysis for the active extrusion of substances against a concentration gradient [34,35]. Depending on the cells and tissues, they regulate the absorption or the excretion of endogenous substances (e.g., hormones, bile salts) and xenobiotics. Generally, ABC transporters limit drug absorption, distribution, and interaction with intracellular targets and facilitate drug/metabolite elimination. Several studies showed that working together with drug-metabolizing enzymes contributed to governing drug efficacy. Additionally, in vivo and in vitro studies suggest that this metabolic cooperation frequently results in DDIs [36]. As presented for drug-metabolizing enzymes, simultaneous exposure to drugs, genetic variability, and other endogenous and exogenous factors can impact ABC transporter expression and function. From a pharmacological point of view, the most studied and characterized members are P-glycoprotein (P-gp, MDR1, ABCB1), multidrug resistance-associated proteins (MRPs, ABCCs), and breast cancer resistance protein (BCRP, ABCG2) [37]. P-gp mediates the efflux of a wide variety of substrates, a large number of which undergo cytochrome P450 (CYP3A4) metabolism. In addition, since its first discovery, it has been well-established that P-gp mediates resistance to a broad spectrum of chemotherapeutic agents [38]. More importantly, given its wide tissue distribution and relation with CYP450 enzymes, P-gp is frequently involved in DDIs. In this regard, the transport of the P-gp substrate digoxin is frequently evaluated in in vitro and in vivo assays to predict the potential of new molecular entities to interact with P-gp [36,39]. MRP2 (multidrug-resistance-associated protein 2) is highly expressed at the liver canalicular membrane, the brush border (apical) membrane of the enterocytes, and the apical membrane of the kidney proximal tubule cells, where it mediates exogenous elimination into bile, intestinal duct, and urine, respectively [40]. Most MRP2 substrates are glucuronide, glutathione, and sulfate conjugates, as well as unconjugated drugs, like vinca alkaloids. Different from P-gp, MRP2 is, in addition, highly involved in the enterohepatic circulation of substrates, such as bile acids. Additionally, BCRP, which was originally found in breast cancer cells, is expressed in the liver, kidney, intestine, brain, and placenta, among other tissues. Moreover, due to its expression on the membrane of tumor cells, BCRP frequently mediates tumor resistance against anticancer drugs [41,42]. For instance, cells with BCRP up-regulation exhibit increased resistance to drugs, such as methotrexate, mitoxantrone, topotecan, and irinotecan. BCRP also regulates the traffic of endogenous molecules, such as folic acid and uric acid. In this regard, it has been documented that alteration in BCRP function may underlie gout disease [43].

Altogether, variations in the expression and function of drug transporters, as well as DMEs, caused, for example, by interindividual variability or drugs, can frequently account for DDIs, therapy failure, and toxicity. Therefore, the assessment of the enzyme and transporter expression, together with other clinical parameters, appears to be a promising tool for providing the best therapy for each individual patient.

### 1.2. Extracellular Vesicles

Extracellular vesicles (EVs) are nanoparticles released by almost all cells of the organism to the extracellular space. They have been categorized into exosomes, microvesicles (also known as microparticles or ectosomes), and apoptotic bodies, depending on their biogenesis and size range. Exosomes originate from membrane endosomal compartments after the endocytosis of fragments of the plasma membrane and are secreted to the extracellular space and biological fluids as particles with a diameter between 30 and 150 nm [44,45]. During endocytosis, membranes and soluble proteins are loaded into the endocytic compartments. Proteins from the Golgi complex are loaded into the endosomes, as well [44]. Exosomes are characterized by the presence of tetraspanins (e.g., CD9, CD63, CD81) and other proteins, such as flotillin and TSG101 (tumor susceptibility gene 101) [44]. Microvesicles exhibit a wider size range (up to 1000 nm) and are released by outward budding of the plasma membrane. Apoptotic bodies have a specialized function during programmed cell death and are beyond the scope of this manuscript [45]. So far, EVs have been described as mediators of cell–cell communication in physiological and pathophysiological situations. Participation of EVs in processes such as the immune response and reproduction has been described. Also, signaling by EVs has been associated with the pathogenesis of metabolic disorders and cancer [44]. In this article, we will use the term EVs, following the recommendations and guidelines of the International Society for Extracellular Vesicles [46], to refer to exosomes and microvesicles, as most isolation and analysis methods cannot clearly distinguish both types of EVs. 

**Figure 1:**
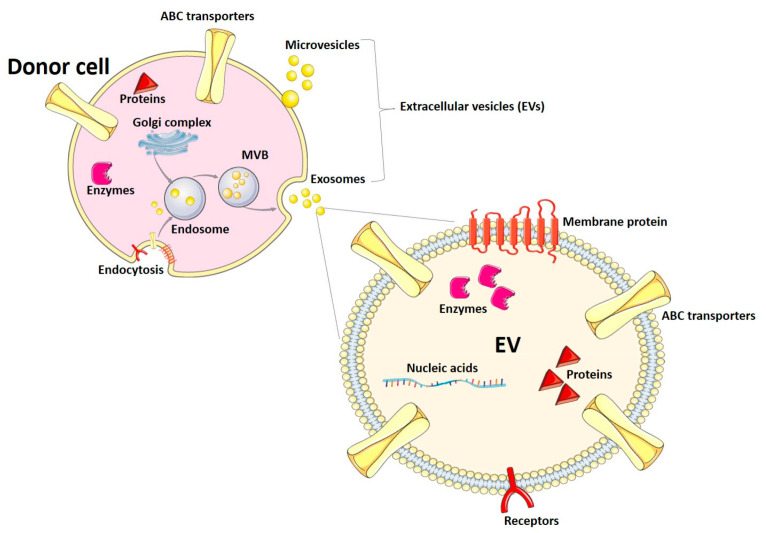
Summary of EV biogenesis mechanisms. Depicted are the mechanisms of exosome and microvesicle biogenesis, as well as the main components of the EV cargo. Abbreviations: EV: extracellular vesicle; MVB: multivesicular body. The figure was partially created using pictures from Servier Medical Art by Servier (Suresnes, France), licensed under a Creative Commons Attribution 3.0 Unported License.

Most of the EV cargo consists of proteins, nucleic acids (e.g., mRNAs, micro RNAs, other non-coding RNAs), lipids, and metabolites [45]. The mechanisms of EV biogenesis and the different components of the EV cargo are summarized in Figure 1. Since the vesicle composition usually reflects the cargo of the cells of origin, EVs might have different applications in diagnosis. In fact, their detection and analysis in biological fluids (e.g., blood, urine, breast milk) can provide useful information on the conditions of the tissue and organ of origin, while avoiding invasive procedures, like biopsies. In this article, we will review the use and potential of EVs as a tool to characterize the expression of drug-metabolizing enzymes and transporter proteins, which are key factors underlying drug ADME and DDIs. EV-based approaches could help adjust pharmacotherapy schemes and aid physicians to find the best personalized treatment for each individual patient.

## 2. EVs as Biomarkers of Drug Metabolism

### 2.1. Phase I

#### 2.1.1. Presence of Phase I-DME in EVs

Several studies have demonstrated the presence of phase I enzymes in EVs isolated from biological fluids, as well as EVs from cells cultured in vitro. For instance, DMEs, together with EV markers and subcellular organelle markers, have been found in EVs collected from the culture medium of sandwich-cultured human hepatocytes. In particular, CYP3A5, but also alcohol dehydrogenases (ADH1A, -1B, -1C), aldehyde dehydrogenase (ALDH1A1), aldehyde oxidase (AO), and carboxylesterase (CES1) have been detected by tandem liquid chromatography–mass spectrometry (LC-MS/MS) [47]. Similar findings were obtained in primary rat hepatocyte-derived EVs, where the presence of Cyp2A1, Cyp2A2, Cyp2B3, Cyp2C11, Cyp2D1, Cyp2D3, Cyp2D10, Cyp2D18, and CypD26 was described also by applying LC-MS/MS analysis [48]. In another study, the presence of phase I enzymes in EVs from human plasma has been reported [49]. Here, the expression of four CYP isoenzymes (i.e., CYP1B1, CYP2A6, CYP2E1, and CYP3A4) has been demonstrated at the mRNA level by qRT-PCR and at the protein level by western blot. Notably, reproducible findings were observed for two different plasma donors, with the exception of CYP3A4 expression, which was detected in only one of the donors. Interestingly, the abundance of CYP2E1 mRNA was around 500-fold higher than the other CYPs. CYP1A1 was only detected at the protein level, while CYP2C9 and CYP2D6 were neither detected at the mRNA nor the protein level. Furthermore, plasma EVs showed higher protein levels of CYP2E1 compared to immortalized hepatocytes (HepaRG cells), monocytes (U937 cells), and their derived EVs. Also, the enzymatic activity of CYP2E1 and CYP3A4 present in plasma-derived EVs was determined using the fluorescence-based Vivid^®^ assay. Both isoenzymes displayed detectable activity, thus proving that EVs carry active CYPs. Unfortunately, no comparison between the enzyme expression or activity in the EVs and the tissues of origin was performed [49]. In another study, the presence of CYP1A2, CYP2C8, CYP2C9, CYP2E1, and CYP3A4 mRNA was demonstrated in human plasma EVs. Similarly, CYP2B6, CYP2C8, CYP2C9, CYP2C19, CYP2D6, CYP2E1, CYP2J2, CYP3A4, and CYP3A5 were detected at the protein level [50]. Altogether, these findings confirm the presence of phase I enzymes in EVs and suggest that packaging these proteins is a well-regulated and, importantly, a selective process, as an enrichment of particular CYPs in the EVs is partially observed. Therefore, changes in the physiology and pathophysiology of the cell of origin may affect the sorting of proteins into the EVs. Furthermore, the presence of functional phase I enzymes in the vesicles bears significant potential in biomarker research, since expression data might not always reflect the enzymatic activity.

#### 2.1.2. EVs as Dynamic Surrogates for Phase I Enzymes

Further studies have addressed changes in the expression levels of CYPs in EVs after different chemical and pharmacological stimuli and, in some cases, with the intent to ascertain whether they reflect changes occurring in the relevant tissues [50,51,52,53,54,55,56]. In particular, one study investigated changes in the CYP-related EV cargo after exposure to galactosamine (galN) and *Escherichia coli*-derived lipopolysaccharide (LPS). In fact, qualitative and quantitative assays demonstrated that the composition and proteome of EVs from primary rat hepatocytes may considerably vary in response to these hepatotoxins. Treatment with galN resulted in the up-regulation of Cyp2A1, Cyp2B2, Cyp2B3, Cyp2C11, Cyp2C23, Cyp2D10, and Cyp2D18. Exposure to LPS decreased the levels of Cyp2D4 in the EVs. If a correlation between the levels of the different enzymes in the EVs and the liver can be established, it would support the potential of EVs to predict changes in drug metabolism under situations of hepatic injury. Unfortunately, no comparison of the hepatic CYP levels in vivo and in EVs was performed [51]. In another study, an increase in the protein levels of CYP1A1/2, CYP2A6, CYP2E1, and CYP4B was demonstrated by western blotting in serum EVs from alcoholic individuals with respect to EVs from healthy volunteers. In addition, increased levels of Cyp1A1/2, Cyp2A3, Cyp2E1, and Cyp4B were observed in circulating EVs from rats treated with ethanol. Importantly, the same trend was observed in liver lysates, which supports the potential of EVs as surrogate markers for tissue changes in the levels of CYPs induced by ethanol administration. It should also be noted that the absence of Cyp2E1, as in the case of Cyp2E1-null mice, led to different changes in the regulation of the levels of Cyps in the liver and the EVs. In particular, the up-regulation of Cyp1A1/2 by ethanol in the liver and EVs was not observed in mice lacking Cyp2E1, whereas a stronger up-regulation of Cyp4B was reported in the EVs, but not in the liver [52]. Overall, these findings highlight the potential of EVs as surrogates for the expression of CYPs, and also in response to toxic stimuli. However, previous studies also point to a large number of factors and conditions, which may affect the loading of specific proteins in the vesicles and, in this way, their use as surrogates.

In another study, CYP2E1 was detected in human plasma EVs. Additionally, immortalized hepatocytes were treated with CYP2E1-containing plasma EVs and different concentrations of ethanol or acetaminophen, such as two CYP2E1 substrates. In general, the addition of EVs increased the cytotoxicity of the ethanol and acetaminophen treatment due to the formation of toxic metabolites by CYP2E1. The relevance of CYP2E1 within the EV cargo was confirmed using inhibitors and siRNAs [53]. Interestingly, CYP2E1 expression and activity may be estimated by using EVs but without a direct measurement of the enzyme levels. Instead, the levels of a regulatory miRNA in EVs might be used. In fact, following acetaminophen-induced liver injury, the transfer of miR-122 from the hepatocytes to the kidney via EVs resulted in the down-regulation of renal CYP2E1 expression and activity. This reduced the renal toxicity of cisplatin (which depends on the CYP2E1 activity). These findings were confirmed in vitro, where the uptake of these particular serum EVs decreased CYP2E1 in renal proximal tubule cells via miR-122. The involvement of the liver as donor tissue for the EVs was confirmed using a tissue-specific Dicer knock-out (i.e., impaired miRNA production) [54]. Altogether, these findings point also to the potential of miRNA levels in EVs as surrogates for enzyme expression in certain tissues. Nevertheless, the applicability of this approach would need to be confirmed for each particular case.

Although several studies have identified the presence of phase I enzymes in EVs, their use and potential as markers of enzymatic induction by drugs and in relation to drug metabolism have only been investigated in a few studies. In this regard, Rowland et. al. [50] showed a clear increase in the mRNA and protein levels of CYP3A4 in human plasma EVs from individuals treated with the well-known inducer rifampicin (300 mg/day, 7 days, p.o.). An expected increase in the CYP3A4 activity in the EVs by rifampicin was observed, as well [50]. Additional experiments determined the correlation between the apparent oral clearance (CL/F) of midazolam, used as a probe substrate, and CYP3A4 levels in plasma EVs (i.e., mRNA and protein expression and ex vivo activity) pre- and post-rifampicin exposure. These relations were measured in terms of the R^2^ correlation coefficient. Results were R^2^ = 0.787 for mRNA expression, 0.905 for protein expression, and 0.832 for ex vivo activity, which are indicative of a positive correlation between CYP3A4 expression and activity in the EVs and the clearance of a model substrate [50]. 

In line with these findings, enzymatic induction analysis was also performed by Rodrigues and colleagues [55]. Liver-derived EVs and non-liver-derived EVs were isolated from the serum of a cohort of healthy men treated with midazolam and dextromethorphan (probe drugs for CYP3A4/5 and CYP2D6, respectively) prior to and after rifampicin exposure. Two rifampicin dosing protocols were tested (300 mg/day for 7 days and 600 mg/day for 14 days). After both treatments, a clear decrease in midazolam plasma AUC was observed, as expected, compared to the AUC curve obtained before RIF treatment. Interestingly, both treatments with rifampicin also led to an increase in CYP3A4 protein levels in liver and non-liver EVs [55]. The same group also described the effects on the EV cargo produced by CYP3A4 induction by modafinil (400 mg day/14 days). Although a correlation between basal CYP3A4 protein levels in liver EVs and the ratio 4β-hydroxycholesterol/cholesterol (i.e., a marker of CYP3A4 activity) in plasma was observed, this association was not observed after induction [56]. Also, CYP2D6 levels in liver EVs correlated with the basal enzyme activity in vivo (determined by measurement of dextromethorphan O-demethylation). However, exposure to rifampicin did not increase CYP2D6 expression or enzymatic activity in liver EVs, even if the dextromethorphan metabolite formation in vivo increased [55]. These observations clearly highlight the differences between the use of EVs as surrogates for different DMEs. While the expression of an enzyme in the EVs and its changes after the administration of a particular inductor may highly resemble the expression changes in vivo, as observed for rifampicin and CYP3A4 [50], the expression of another enzyme in the EVs may not necessarily reproduce the changes in metabolite formation in vivo, as demonstrated for rifampicin and CYP2D6-dependent metabolism. Similarly, the use of EVs as surrogates for the changes in the enzyme expression by exposure to a particular inductor does not guarantee their use for other different inductors (i.e., EVs do not reflect the changes in CYP3A4 activity in vivo by modafinil). The cases where phase I DMEs have been detected, and eventually regulated, in EVs, are summarized in Table 1. 

#### 2.1.3. Use of EVs for Patient Stratification

In addition to the complexity of the use of EVs to predict the extent of DME regulation by diverse stimuli or perpetrators in a dynamic way, EVs may still be used as a tool to predict interindividual variability in drug exposure by monitoring the basal levels of DMEs. In a recent study, a novel pharmacological test based on the use of liquid biopsy measurement as an alternative to genotyping has been developed. The authors described the correlation between cell-free RNA (cfRNA) from CYP3A4, CYP3A5, CYP2D6, CYP2C9, CYP1A2, CYP2A6, CYP2E1, and CYP2C19 in plasma EVs and the corresponding protein levels in the liver. Interestingly, this study integrated an estimation of the EV shedding from the tissue into the plasma as a factor modifying the measurements of DMEs in plasma EVs. Results from an ROC (receiver operator characteristic) analysis pointed to the potential of this EV-based approach toward stratification of the individuals in fast metabolizers, intermediate metabolizers, and slow metabolizers [57]. The suitability of this method was further confirmed in a second study from the same group, in which a correlation between cfRNA of four CYP enzymes (CYP1A2, CYP2B6, CYP2C9, and CYP3A) in plasma EVs and their activities in patients with cardiovascular disease was established [58]. This could be a starting point for the use of EV analysis for patient stratification prior to drug administration either in clinical practice or clinical trials. However, liquid biopsy predictions formulated only on the cfRNA quantification may not represent a reliable alternative to therapeutic drug monitoring (TDM) yet, given that CYP activity is influenced not only by genetic variation and the regulation of the mRNA expression but also by post-transcriptional modifications, which may not be reflected in the amount of cfRNA loaded into the EVs [59].

### 2.2. Phase II 

Unlike phase I enzymes, the presence of phase II conjugating enzymes in EVs and their regulation in response to changes in the tissues of origin has not been extensively studied. Furthermore, with a few exceptions, most of the evidence available so far relies on the use of in vitro and/or animal models. For instance, a proteomics analysis of EVs from rat hepatocyte primary culture identified the presence of Gstm1, Sult1a1, Ugt1a6, Ugt1a8, Ugt2b1, Ugt2b2, Ugt2b3, and Ugt2b5. Notably, neither the hepatocytes nor the animals prior to the cell collection were subject to any treatment [48]. In human hepatocytes cultured in a sandwich, a model slightly closer to a physiological condition, the presence of SULT2A1 was detected in EVs isolated from the supernatant. Here, the peak of expression in the EVs was achieved at 48 h of culture and significantly decreased when the EVs were collected after 72 h of culture, thus highlighting the relevance of culture and collection time [47]. In a further study carried out in rat primary hepatocytes obtained from animals treated with the hepatotoxin galN, the presence and increase in the protein levels of Ugt1A1, Ugt1A2, Ugt1A3, Ugt1A6, Ugt1A7, Ugt1A8, Ugt2B17, and Ugt2B37 in EVs was demonstrated through proteomics analysis. The data also showed the up-regulation of Gstm1, Sult1E1, and Sult1E3. Furthermore, Sult1E2 and Ugt2B2 were up-regulated in EVs from primary hepatocytes not only from rats treated with galN but also from animals treated with bacterial LPS. Both treatments also led to a decrease in the content of Gstm2 in the EVs. Finally, an increase in Gsta1 and Gstm4 was observed in hepatocyte-derived EVs from rats treated with LPS [51]. 

So far, the most significant contribution for the use of phase II enzyme expression in EVs as biomarkers of tissue metabolic activity was provided by Achour et al., [57]. In this study, involving liver cancer patients, a significant correlation between the mRNA of UGT1A1, UGT1A9, UGT2B4, and UGT2B7 in plasma EVs and the protein expression of these enzymes in liver tissue was demonstrated. Notably, as previously described for phase I enzymes (see Section 2.1.3), the authors normalized the mRNA content in the EVs to a shedding factor, based on the quantification of liver-specific markers in plasma EVs, to account for interindividual variability in the release of tissue-specific EVs to the plasma. This approach resulted in a significant improvement in the correlation between plasma EVs and liver tissue [57]. Additionally, the same study provided evidence on the presence of the mRNA of several other phase II enzymes in plasma EVs (UGT1A3, UGT1A4, UGT1A8, UGT1A10, UGT2A3, UGT2B10, UGT2B11, UGT2B15, UGT2B17, UGT3A1, UGT3A2, GSTA1, GSTA2, GSTM1, GSTM2, GSTM4, GSTM5, GSTT1, SULT1A1, SULT1A2, SULT1A4, SULT1B1, SULT1C2, SULT1E1, and SULT2A1) [57]. Along the same line, Rowland et al. identified UGT1A1, UGT1A9, UGT2B4, UGT2B7, UGT2B10, and UGT2B15 at the mRNA level and UGT1A1, UGT1A3, UGT1A4, UGT1A6, UGT1A9, UGT2B4, UGT2B7, UGT2B10, and UGT2B15 at the protein level in EVs from human plasma [50]. Although a correlation analysis between the levels of these mRNAs in plasma EVs and the corresponding protein levels in the tissues of origin were not performed for any of the detected mRNAs, these findings, confirming the presence of the enzyme messengers in the plasma EVs, represent the first step toward analyzing their use as surrogates of phase II drug metabolism.

In healthy volunteers, proteomics analysis of urinary EVs isolated by ultracentrifugation identified the presence of GSTA1, GSTA2, GSTM2, GSTM3, GSTM5, and GSTP1 [60]. Along the same line, another proteomics study detected GSTA1, GSTA2, GSTA3, and GSTM3 in the same type of vesicles [61]. A further study from the same group confirmed the presence of GSTA1 and also identified GSTM2 and GSTP1 in urinary EVs isolated by ultracentrifugation [62]. Surprisingly, no members of the UGT families were detected in urinary EVs, despite their relevant expression and activity in renal tissue [63]. 

Altogether, the studies on phase II enzymes point to the representation of the main phase II enzyme types in plasma EVs, where in the case of UGTs, a correlation with the content in the tissue of origin was established [57], while on the contrary, in urinary EVs, a higher potential for GST determination has been observed (Table 2). In the case of circulating EVs and urinary EVs it still has to be demonstrated whether the EVs also reproduce changes in the enzyme expression upon exposure to regulatory substances (e.g., inducers).

## 3. EVs as Biomarkers of Drug Transport

### 3.1. Uptake Transporters

A proteomics study in EVs from primary rat hepatocytes identified the presence of Oatp1A3 and Oatp1A4 [48]. Furthermore, in the frame of clinical trials, the presence of different uptake transporters was demonstrated. One of the most advanced studies of the clinical application of EVs as surrogates for uptake transporter expression identified OATP1B1 mRNA in liver-derived plasma EVs. Moreover, evidence of a correlation between mRNA levels in the EVs and hepatic protein expression was provided [57]. These findings highlight the potential of EVs as a tool to estimate the transporter expression in the tissue of origin. However, it is still unclear to what extent these measurements can be correlated with the pharmacokinetics of OATP1B1 substrates. Another study investigated whether the expression levels of OATP1B1 and OATP1B3 in plasma EVs are affected by treatment with the transporter inducer rifampicin. In fact, neither 300 mg of rifampicin for 7 days nor 600 mg of rifampicin for 14 days modified the content of OATP1B1 or OATP1B3 in the EVs collected from the plasma of healthy volunteers [55]. While in principle, the detection of OATP1B3 in plasma EVs, as well as the confirmation of OATP1B1 expression in another study using a different type of sample, constitute a relevant advance in the field, data on the induction of both OATPs by rifampicin in the liver is still not conclusive [64]. Hence, due to the lack of a clear induction protocol, it is still unclear whether plasma EVs can reflect changes in the transporter expression in vivo. 

Further studies have analyzed the presence of SLC transporters in human urinary EVs. A proteomics analysis identified OATP4C1 (SLCO4C1), OAT1 (SLC22A6), OAT3 (SLC22A8), OAT4 (SLC22A11), OAT10 (SLC22A13), OCT2 (SLC22A2), and OCTN2 (SLC22A5) [62]. Additionally, the presence of Oatp1A1 (Slco1A1) was demonstrated in urine from rats with bilateral ureteral obstruction (i.e., model of obstructive nephropathy). The protocol used by the authors was based on the western blot analysis of urine samples after clearing the urine by centrifugation at 3000 g [65]. In this regard, although membrane proteins carried by urinary EVs have been already detected by antibody-based methods in urinary samples subject to a similar preparation (i.e., without prior EV enrichment) [66], the specific confirmation of the localization of OATP1A1 in EVs is still missing (e.g., by colocalization with EV markers) [65]. Along the same line, OAT1, OAT3, and OAT4 were demonstrated in urinary EVs from patients with acute kidney injury. While OAT1 and OAT3 levels in EVs peaked concomitantly with the peak of the injury, OAT4 exhibited a decrease at the most severe stage of the injury and increased after renal improvement. Notably, similarly to the previous case [65], although the membrane preparation protocol used is compatible with enrichment in urinary EVs, no positive markers (e.g., tetraspanins) to confirm the presence of exosomes or other EV populations were analyzed [67].

Overall, the evidence obtained so far points to the presence of uptake transporters in circulating as well as urinary EVs and, eventually, the correlation between the amount of transporter in the EVs and the tissues of origin. This evidence is summarized in Table 3. Future research should be aimed at elucidating whether the transporter content in the EVs reflects changes in the transporter expression in the tissues of origin, for example by drugs. 

### 3.2. Efflux Transporters

#### 3.2.1. Plasma and Serum EVs

Profiling the cargo of plasma EVs as surrogates for the ABC transporter expression in the tissues of origin and their changes in response, for example, to pharmacological stimuli, could represent a strategy to estimate drug disposition, organ excretion capability, and interindividual variability in drug response. Several studies confirmed the presence of efflux transporters in plasma and serum EVs, and a few of them also investigated their role as potential markers for drug transport (Table 4). Particularly, they focused on the use of EVs to characterize interindividual variability by monitoring transporter expression. For instance, in one work, it has been hypothesized that miR-328 carried by intestinal EVs present in the plasma can be proposed as a biomarker to predict intestinal BCRP functionality. In fact, BCRP expression and activity in human tissues can be negatively modulated by miRNA-328 [68]. An open-label, non-randomized, single-arm clinical study involving 33 healthy volunteers estimated BCRP activity through the establishment of a correlation between miR-328 plasma levels in intestine-derived EVs and the AUC of sulfasalazine (SASP), a known BCRP substrate. In particular, intestine-derived EVs were isolated from the whole plasma EVs by using an antibody against human glycoprotein A33 (anti-GPA33), a protein specifically enriched in the human intestine. Moreover, intestine-specific miRNAs (miR-192 and miR-215) have been utilized to successfully distinguish intestine-derived EVs from general plasma- and hepatocyte-derived EVs. The presence of miR-328 in immunoprecipitated particles was later confirmed by qRT-PCR analysis, and a reduction in miR-328 levels in different conditions (detergent or RNAse A) was consistent with miR-328 packaging in the intestine-derived EVs. The authors observed that the levels of miR-328 in intestine-derived EVs correlated with the AUC of SASP, suggesting that subjects with higher miR-328 levels are characterized by higher SASP bioavailability, most likely due to lower intestinal BCRP functionality [68]. Although the present work clearly determined an association between intestine-derived EVs and BCRP activity, it is important to note that BCRP activity may be affected by a few additional miRNAs [69], polymorphisms, and other regulatory factors. Thus, it is not clear whether the regulation of BCRP by inducers, such as PXR activators, can be assessed by measuring miR-328 levels in EVs. Furthermore, some BCRP substrates may also be transported by other members of the ABC superfamily [37]. Further studies with confirmation cohorts, as well as other transporter substrates and during exposure to other drugs, may aid in elucidating the full potential of this miRNA for clinical application. 

In another study, a new approach for patient stratification that replaces or complements traditional techniques (e.g., genotype analysis) was tested. In this context, a measurement of P-gp, BCRP, and MRP2 in plasma EVs has been performed. After the extraction and analysis of plasma EVs, a strong correlation between the mRNA levels of transporter transcripts in liver-derived EVs in plasma and hepatic protein levels has been found [57]. The established relation is underlined by the results obtained from ROC analysis that combined with data on DMEs demonstrate their utility in drug dose optimization based on patients’ characteristics. This same approach was then applied by the same researchers in a second study conducted on patients with cardiovascular disease. P-gp mRNA expression in plasma EVs was measured and a correlation with the transporter activity, determined based on the plasma concentration and AUC of fexofenadine, was established [58]. As already applied by the authors for the analysis of phase I, II, and uptake transporters in EVs, the P-gp expression in the EVs was corrected by a factor representative of the shedding of EVs by the liver to the circulation. 

In addition to the advantages of transporter expression and quantification in EVs to estimate drug distribution and systemic clearance, analysis of ABC transporters in EVs may be used to predict cancer drug resistance and therapeutic response. In this regard, it has been demonstrated that P-gp levels in serum EVs from prostate cancer patients resistant to docetaxel-based chemotherapy were higher than in serum EVs from therapy-responsive patients [70]. 

Altogether, several studies demonstrated the presence of drug transporters in circulating EVs in health and disease. Furthermore, experimental evidence obtained in clinical trials supports the potential of EVs to stratify individuals based on their transporter expression. Conversely, further research is required to elucidate whether EVs can also be used as dynamic surrogates, which not only reflect the basal expression of the transporters but also deliver time-dependent information about changes in the transporter expression; for example, due to exposure to inducers or pathophysiological alterations.

#### 3.2.2. Urinary EVs

Urine-derived EVs have been investigated as a potential source of biomarkers for more than a decade. For instance, it has been postulated that aquaporin 2 (AQP2), a membrane channel associated with water balance disorders, and other apical transporters are released in urinary EVs. This prediction was verified by performing an immunoelectron microscopy analysis (ImmunoGold), revealing that the vesicle orientation (cytoplasmic-side inward) was compatible with exosome formation and presence. In fact, urinary exosomes derive from multivesicular body (MVB) fusion with the apical plasma membrane of renal epithelial cells through an invagination process. Also, the size distribution of urinary EVs highly matched with exosome size criteria (<100 nm in diameter) [61]. The proteomics analysis of these urinary EVs detected the presence of a variety of proteins involved in different cellular functions and processes. Among them, P-gp was observed [61]. Similar results were obtained in two other works, confirming the presence of a large number of proteins mostly involved in solute and water transport in human urinary EVs. In terms of drug transporters, P-gp was also detected in both studies [60,62]. Although these studies provided experimental evidence on the loading of P-gp into urinary EVs, they have not investigated their potential as biomarkers for ADME and/or DDIs. Firstly, one of the aims of the previously mentioned studies was to expand the existent human urinary EVs database through the validation of a sensitive and precise method for their identification and analysis. Secondly, possible interference may be generated during sample management since standard protocols for the collection, processing, and storage of urine samples are still lacking. Finally, the normalization strategies for protein expression data in urinary EVs still need to be established.

## 4. Future Perspectives and Challenges

**Figure 2:**
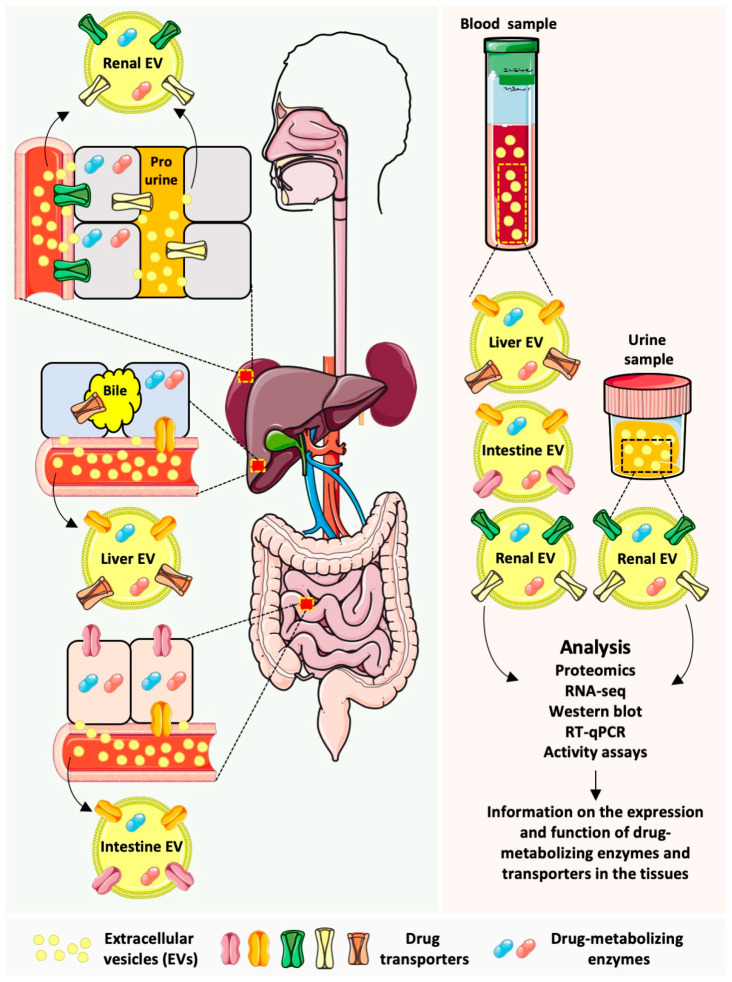
EVs as surrogates of DMEs and drug transporter expression and activity. Left panel: hepatocytes, enterocytes, and renal tubular cells constitute the major cell types involved in drug absorption, metabolism, and excretion. DME enzymes mediate the biotransformation of drugs, while drug transporters are membrane proteins that mediate the drug uptake into the cell or the efflux to the extracellular space (bile in the case of hepatocytes, intestinal lumen in the case of enterocytes, and pro-urine in the case of renal tubular cells; the basolateral efflux to the blood can also take place). All these cells release EVs carrying DMEs and drug transporters to the blood and, in the case of renal cells, also the urine. The cargo of the EVs may reflect the composition of the cell of origin. Right panel: intestinal and hepatic EVs can be isolated from blood, while renal EVs can be isolated from blood and urine. EVs can be subjected to different analytical methods with the aim of investigating the DME and transporter expression. Also, the activity of DMEs in EVs can be determined. This information may reflect the expression and activity in the tissues of origin and, therefore, contribute to estimating the drug-metabolizing and excretion capacity of an individual in a minimally invasive way. The figure was created using pictures from Servier Medical Art by Servier (Suresnes, France), licensed under a Creative Commons Attribution 3.0 Unported License.

In the previous sections, we described the presence of several drug-metabolizing enzymes and transporter proteins in EVs (Figure 2). While some data results from in vitro experimentation, significant evidence has also been obtained in vivo in the frame of clinical trials. However, the way toward the application of EVs as surrogates for the expression in vivo and, moreover, for the pharmacokinetics of DME or transporter substrates is still long and uncertain. In this regard, while the correlation between the expression in the tissue of origin and EVs has been demonstrated in different cohorts for different proteins, it is still unclear to what extent EVs may reproduce the changes in the protein expression in the tissues of origin. In this regard, research on phase I enzymes is a step forward, with the confirmation of CYP3A4 regulation in EVs and even, in some cases, its correlation with pharmacokinetic parameters [50,55,56]. However, even in these cases, the correlation between phase I enzymes in the EVs and their activity in vivo does not apply to all substrates investigated. Moreover, data obtained after exposure to inducers should be cautiously analyzed. In general, the loading of proteins into the EVs is a complex process consisting of different independent mechanisms. Therefore, it is unclear whether inductions in the tissue of origin may always lead to changes in the EV cargo, irrespective of the inducer compound. Here, further studies investigating the effect of other inductors different from rifampicin and inductors acting through other pathways or at other levels of regulation (e.g., translational or post-translational) could provide more evidence for the use of EVs as biomarkers. Furthermore, the timeframe is an important factor that should be considered. Currently, most evidence has been obtained at one or a few timepoints after addition or treatment with the inducer. In this regard, it is necessary to know the time required for the onset of changes in tissue expression at the EV level. In addition, another question to be solved is whether EVs can dynamically reflect small changes in the protein expression in the tissues of origin. For this purpose, optimization and standardization of isolation and highly sensitive and specific analytical methods are urgently required. 

Most EV isolation protocols rely on the execution of several purification steps, which may not only result in selective damage of the EV cargo (e.g., surface proteins) but also a decrease in the EV protein yield. In relation to this, analytical methods, which can be directly applied to the biological sample, would be an important contribution toward eliminating processing-related biases. In this regard, fluorescent nanoparticle tracking analysis (NTA) has been successfully applied for the detection of AQP2 in urinary EVs obtained following minimal urine sample processing. Importantly, thanks to a soft preincubation with a detergent, even the detection of internal epitopes (e.g., not located on the external side of the EV) has been achieved [66]. Along the same line, the recently developed EV Quant technology may also be used for the analysis of surface proteins in urinary EVs without extensive sample processing [66]. It should be noted, however, that these methodologies may not be applicable, at least in their current state, to more complex and protein-rich samples, such as plasma. 

Once accurate data on the protein expression in EVs have been obtained, data normalization constitutes the next challenge. In the case of urinary EVs, urinary creatinine has been reported to correlate with the concentration of vesicles in the sample [66]. Therefore, in this case, a normalization of the enzyme or transporter expression to the urinary creatinine may be considered. Also, the high abundance of renal-derived EVs in the urine and the comparatively lower expression of DMEs and transporters in non-renal urinary tract EVs facilitate data interpretation. On the contrary, plasma EVs bear the challenge not only of data normalization but also tissue specificity. In this regard, the correction of the EV protein levels by a shedding factor, accounting for the EV release from a specific tissue into the circulation, appears as the most promising strategy [57,58]. While this strategy was successfully applied for the analysis of liver-derived EVs, specific EV markers and their application for other tissues still have to be investigated. Another alternative is the direct isolation of tissue-specific EVs from circulation [55,68]. While this approach has been successfully applied for liver [55] and intestinal EVs [68], specific markers for other pharmacologically relevant tissues (e.g., brain vessel endothelium) should be identified. However, in this case, considering that some individuals may release more EVs into circulation than others, it should be confirmed whether normalization to total (tissue-specific) EV protein represents the most accurate approach.

## 5. Conclusions

In the past sections, we have described different findings supporting the use of EV cargo as a surrogate to predict the expression and eventual activity of DMEs and drug transporters. Even with the current experimental limitations related to EV isolation, analysis, and normalization, most evidence, especially related to phase I enzymes, indicates a major potential of EVs to estimate interindividual variability in the enzyme expression and function. Data on phase II enzymes and drug transporters, as well as under situations of up-regulation, has been, so far, less conclusive. Methodological advances and optimization are strongly required in terms of tissue-specific EV isolation, analysis, and data normalization. The improvement in the quality of the data obtained from EVs may lead to the identification of significant correlations in EV versus the tissue of origin for other DME and transporters, as well as dynamically in case of regulation by drugs or pathophysiological situations. If this is finally achieved, then EVs may definitely become a minimal or non-invasive tool to predict drug exposure and, this way, optimize therapy by increasing drug efficacy and safety.

## Figures and Tables

**Table 1:** Phase I DMEs detected in EVs.

Enzyme	Detection Level	Sample	Regulation	References
CYP1A1	Protein Protein Protein	Serum Plasma (rat) Plasma (mice)	Ethanol ↑ Ethanol ↑ Ethanol ↑	[52]
CYP1A2	Protein Protein Protein mRNA, protein	Serum Plasma (rat) Plasma (mice) Plasma	Ethanol ↑ Ethanol ↑ Ethanol ↑ ─	[52] [50,57]
CYP1B1	mRNA, protein	Plasma	─	[49]
CYP2A1	Protein Protein	Culture medium (rat hepatocytes) Culture medium (rat hepatocytes)	galN ↑ ─	[51] [48]
CYP2A2	Protein	Plasma (rat)	Ethanol ↑	[52]
CYP2A3	Protein	Plasma (rat)	Ethanol ↑	[52]
CYP2A5	Protein	Plasma (mice)	Ethanol ↑	[52]
CYP2A6	Protein mRNA, protein	Serum Plasma	Ethanol ↑ ─	[52] [49,57]
CYP2B2	Protein	Culture medium (rat hepatocytes)	galN ↑	[51]
CYP2B3	Protein Protein	Culture medium (rat hepatocytes) Culture medium (rat hepatocytes)	galN ↑ ─	[51] [48]
CYP2B6	Protein	Plasma	─	[50]
CYP2C8	mRNA, protein	Plasma	─	[50]
CYP2C9	mRNA, protein	Plasma	─	[50,57]
CYP2C11	Protein Protein	Culture medium (rat hepatocytes) Culture medium (rat hepatocytes)	galN ↑ ─	[51] [48]
CYP2C19	mRNA, protein	Plasma	─	[50,57]
CYP2C23	Protein	Culture medium (rat hepatocytes)	galN ↑	[51]
CYP2D1	Protein	Culture medium (rat hepatocytes)	─	[48]
CYP2D3	Protein	Culture medium (rat hepatocytes)	─	[48]
CYP2D4	Protein	Culture medium (rat hepatocytes)	LPS ↓	[51]
CYP2D6	mRNA, protein	Plasma	─	[50,55,57]
CYP2D10	Protein Protein	Culture medium (rat hepatocytes) Culture medium (rat hepatocytes)	galN ↑ ─	[51] [48]
CYP2D18	Protein Protein	Culture medium (rat hepatocytes) Culture medium (rat hepatocytes)	galN ↑ ─	[51] [48]
CYP2D26	Protein	Culture medium (rat hepatocytes)	─	[48]
CYP2E1	Protein Protein Protein mRNA, protein	Serum Plasma (rat) Plasma (mice) Plasma	Ethanol ↑ Ethanol ↑ Ethanol ↑ ─	[52] [49,50,57]
CYP2J2	Protein	Plasma	─	[50]
CYP3A4	mRNA, protein, enzymatic activity mRNA, protein	Plasma Plasma	Rifampicin ↑ ─	[50,55,57] [49]
CYP3A5	Protein mRNA, protein	Culture medium (human hepatocytes) Plasma	─ ─	[47] [50,57]
CYP4A	Protein Protein Protein	Serum Plasma (rat) Plasma (mice)	─ ─ ─	[52]
CYP4B	Protein Protein Protein	Serum Plasma (rat) Plasma (mice)	Ethanol↑ Ethanol↑ Ethanol↑	[52]

Summarized are the level at which the enzymes were detected, the type of sample, and if changes in the EV content in response to pharmacological or toxic stimuli occur. Unless otherwise stated, data refer to human samples and EVs. Abbreviations: galN: galactosamine; LPS: lipopolysaccharide; ↑: up-regulation; ↓: down-regulation.

**Table 2:** Phase II drug-metabolizing enzymes detected in EVs.

Enzyme	Detection Level	Sample	Regulation	References
GSTA1	Protein mRNA Protein	Culture medium (rat hepatocytes) Plasma Urine	LPS ↑ ─ ─	[51] [57] [60,61,62]
GSTA2	mRNA Protein	Plasma Urine	─ ─	[57] [60,61]
GSTA3	Protein	Urine	─	[61]
GSTM1	Protein Protein mRNA	Culture medium (rat hepatocytes) Culture medium (rat hepatocytes) Plasma	galN ↑ ─ ─	[51] [48] [57]
GSTM2	Protein mRNA Protein	Culture medium (rat hepatocytes) Plasma Urine	galN ↓ LPS ↓ ─ ─	[51] [57] [60,61]
GSTM3	Protein	Urine	─	[60,61]
GSTM4	Protein mRNA	Culture medium (rat hepatocytes) Plasma	LPS ↑ ─	[51] [57]
GSTM5	mRNA Protein	Plasma Urine	─ ─	[57] [60]
GSTP1	Protein	Urine	─	[60,62]
GSTT1	mRNA	Plasma	─	[57]
SULT1A1	Protein mRNA	Culture medium (rat hepatocytes) Plasma	─ ─	[48] [57]
SULT1A2	mRNA	Plasma	─	[57]
SULT1A4	mRNA	Plasma	─	[57]
SULT1B1	mRNA	Plasma	─	[57]
SULT1C2	mRNA	Plasma	─	[57]
SULT1E1	Protein mRNA	Culture medium (rat hepatocytes) Plasma	galN ↑ ─	[51] [57]
SULT1E2	Protein	Culture medium (rat hepatocytes)	galN ↑ LPS ↑	[51]
SULT1E3	Protein	Culture medium (rat hepatocytes)	galN ↑	[51]
SULT2A1	Protein Protein mRNA	Culture medium (human hepatocytes) Culture medium (rat hepatocytes) Plasma	─ galN ↓ LPS ↓ ─	[47] [51] [57]
UGT1A1	Protein mRNA, protein	Culture medium (rat hepatocytes) Plasma	galN ↑ ─	[51] [50,57]
UGT1A2	Protein Protein	Culture medium (rat hepatocytes) Plasma	galN ↑ ─	[51] [50]
UGT1A3	Protein mRNA, protein	Culture medium (rat hepatocytes) Plasma	galN ↑ ─	[51] [50,57]
UGT1A4	mRNA, protein	Plasma	─	[50,57]
UGT1A6	Protein Protein Protein	Culture medium (rat hepatocytes) Plasma Culture medium (rat hepatocytes)	galN ↑ ─ ─	[51] [50] [48]
UGT1A7	Protein	Culture medium (rat hepatocytes)	galN ↑	[51]
UGT1A8	Protein mRNA Protein	Culture medium (rat hepatocytes) Plasma Culture medium (rat hepatocytes)	galN ↑ ─ ─	[51] [57] [48]
UGT1A9	mRNA, protein	Plasma	─	[50,57]
UGT1A10	mRNA	Plasma	─	[57]
UGT2A3	mRNA	Plasma	─	[57]
UGT2B1	Protein	Culture medium (rat hepatocytes)	─	[48]
UGT2B2	Protein Protein	Culture medium (rat hepatocytes) Culture medium (rat hepatocytes)	─ galN ↑ LPS ↑	[48] [51]
UGT2B3	Protein	Culture medium (rat hepatocytes)	─	[48]
UGT2B4	mRNA, protein	Plasma	─	[50,57]
UGT2B5	Protein	Culture medium (rat hepatocytes)	─	[48]
UGT2B7	mRNA, protein	Plasma	─	[50,57]
UGT2B10	mRNA, protein	Plasma	─	[50,57]
UGT2B11	mRNA	Plasma	─	[57]
UGT2B15	mRNA, protein	Plasma	─	[50,57]
UGT2B17	Protein mRNA	Culture medium (rat hepatocytes) Plasma	galN ↑ ─	[51] [57]
UGT2B37	Protein	Culture medium (rat hepatocytes)	galN ↑	[51]
UGT3A1	mRNA	Plasma	─	[57]
UGT3A2	mRNA	Plasma	─	[57]

Summarized are the level at which the enzymes were detected, the type of sample, and if changes in the EV content in response to pharmacological or toxic stimuli occur. Unless otherwise stated, data refer to human samples and EVs. Abbreviations: galN: galactosamine; GST: glutathione-S-transferase; LPS: lipopolysaccharide; SULT: sulfotransferase; UGT: UDP glucuronosyltransferase; ↑: up-regulation; ↓: down-regulation.

**Table 3:** Drug uptake transporters detected in EVs.

Uptake Transporter	Detection Level	Sample	References
OAT1 (SLC22A6)	Protein	Urine	[62]
OAT3 (SLC22A8)	Protein	Urine	[62]
OAT4 (SLC22A11)	Protein	Urine	[62]
OAT10 (SLC22A13)	Protein	Urine	[62]
OATP1A1 (SLCO1A1)	Protein	Urine (rat)	[65]
OATP1A3 (SLCO1A3)	Protein	Culture medium (rat hepatocytes)	[48]
OATP1A4 (SLCO1A4)	Protein	Culture medium (rat hepatocytes)	[48]
OATP1B1 (SLCO1B1)	mRNA, Protein	Plasma	[55,57]
OATP1B3 (SLCO1B3)	Protein	Plasma	[55]
OATP4C1 (SLCO4C1)	Protein	Urine	[62]
OCT2 (SLC22A2)	Protein	Urine	[62]
OCTN2 (SLC22A5)	Protein	Urine	[62]

Summarized are the level at which the transporters were detected and the type of sample. Unless otherwise stated, data refer to human samples and EVs. No cases of regulation of the transporter levels in EVs were reported. Abbreviations: OAT: organic anion transporter; OATP: organic anion transporter polypeptide; OCT: organic cation transporter.

**Table 4:** Drug efflux transporters detected in EVs.

Efflux Transporter	Detection Level	Sample	References
P-gp (ABCB1)	mRNA Protein	Plasma Urine	[57,58] [60,62]
MRP2 (ABCC2)	mRNA	Plasma	[57]
BCRP (ABCG2)	Regulatory miRNA * mRNA	Plasma Plasma	[68] [57]

Summarized are the level at which the transporters were detected and the type of sample. Unless otherwise stated, data refer to human samples and EVs. No cases of regulation of the transporter levels in EVs were reported. Abbreviations: ABC: ATP-binding cassette; BCRP: breast cancer resistance protein; MRP2: multidrug resistance-associated protein 2; P-gp: P-glycoprotein. * In the case of BCRP, a regulatory miR (miR-328) was detected in plasma EVs, which inversely correlates with the pharmacokinetics of a BCRP substrate in vivo.

## Data Availability

No new data have been generated in this article.
